# Phylo-Spec: a phylogeny-fusion deep learning model advances microbiome status identification

**DOI:** 10.1128/msystems.01453-25

**Published:** 2025-11-24

**Authors:** Junhui Zhang, Fan Meng, Yangyang Sun, Wenfei Xu, Shunyao Wu, Xiaoquan Su

**Affiliations:** 1 College of Computer Science and Technology, Qingdao University 12593, Qingdao, Shandong, China; Universita degli Studi di Verona, Verona, Veronai, Italy

**Keywords:** microbiome, phylogeny, deep learning, disease detection

## Abstract

The human microbiome is crucial for health regulation and disease progression, presenting a valuable opportunity for health state classification. Traditional microbiome-based classification relies on pre-trained machine learning (ML) or deep learning (DL) models, which typically focus on microbial distribution patterns, neglecting the underlying relationships between microbes. As a result, model performance can be significantly affected by data sparsity, misclassified features, or incomplete microbial profiles. To overcome these challenges, we introduce Phylo-Spec, a phylogeny-driven deep learning algorithm that integrates multi-aspect microbial information for improved status recognition. Phylo-Spec fuses convolutional features of microbes within a phylogenetic hierarchy via a bottom-up iteration and significantly alleviates the challenges due to sparse data and inaccurate profiling. Additionally, the model dynamically assigns unclassified species to virtual nodes on the phylogenetic tree based on higher-level taxonomy, minimizing interferences from unclassified species. Phylo-Spec also captures the feature importance via an information gain-based mechanism through the phylogenetic structure propagation, enhancing the interpretability of classification decisions. Phylo-Spec demonstrated superior efficacy in microbiome status classification across two *in silico* synthetic data sets that simulate the aforementioned cases, outperforming existing ML and DL methods. Validation with real-world metagenomic and amplicon data further confirmed the model’s performance in multiple status classification, establishing a powerful framework for microbiome-based health state identification and microbe-disease association. The source code is available at https://github.com/qdu-bioinfo/Phylo-Spec.

**IMPORTANCE:**

The human microbiome profoundly influences health and disease, but current computational tools often overlook the evolutionary relationships among microbes, leading to incomplete or inaccurate interpretations of complex microbial data. Phylo-Spec provides a new way to understand the microbiome by combining microbial abundance, taxonomy, and phylogeny within a unified deep learning framework. This model not only improves the accuracy of health status classification but also highlights key microbial contributors linked to disease. By capturing both microbial diversity and evolutionary context, Phylo-Spec bridges the gap between bioinformatics and biological insight, offering a powerful and interpretable approach for advancing microbiome-based diagnostics and precision medicine.

## INTRODUCTION

Microbial communities inhabit various parts of the human body (1, 2). These microorganisms coexist with their human host, exerting profound effects on health and disease development (3–5). Since the launch of the Human Microbiome Project (), interest has surged in understanding the intricate interactions between the human body and associated microbiomes (6–8). Numerous studies have highlighted the close association between the microbiome and a range of diseases, underscoring its potential in diagnostics (9–11). This is particularly evident in chronic diseases like inflammatory bowel disease (IBD), enteric diarrhea, and type 2 diabetes (T2D) (12–14). For instance, the biodiversity of the gut microbiome is significantly reduced in patients with IBD compared to healthy individuals (15). Such microbial patterns provide a promising basis for distinguishing between healthy and diseased states, offering a potential pathway for precision medicine in microbiome-related diseases.

**Fig 1:**
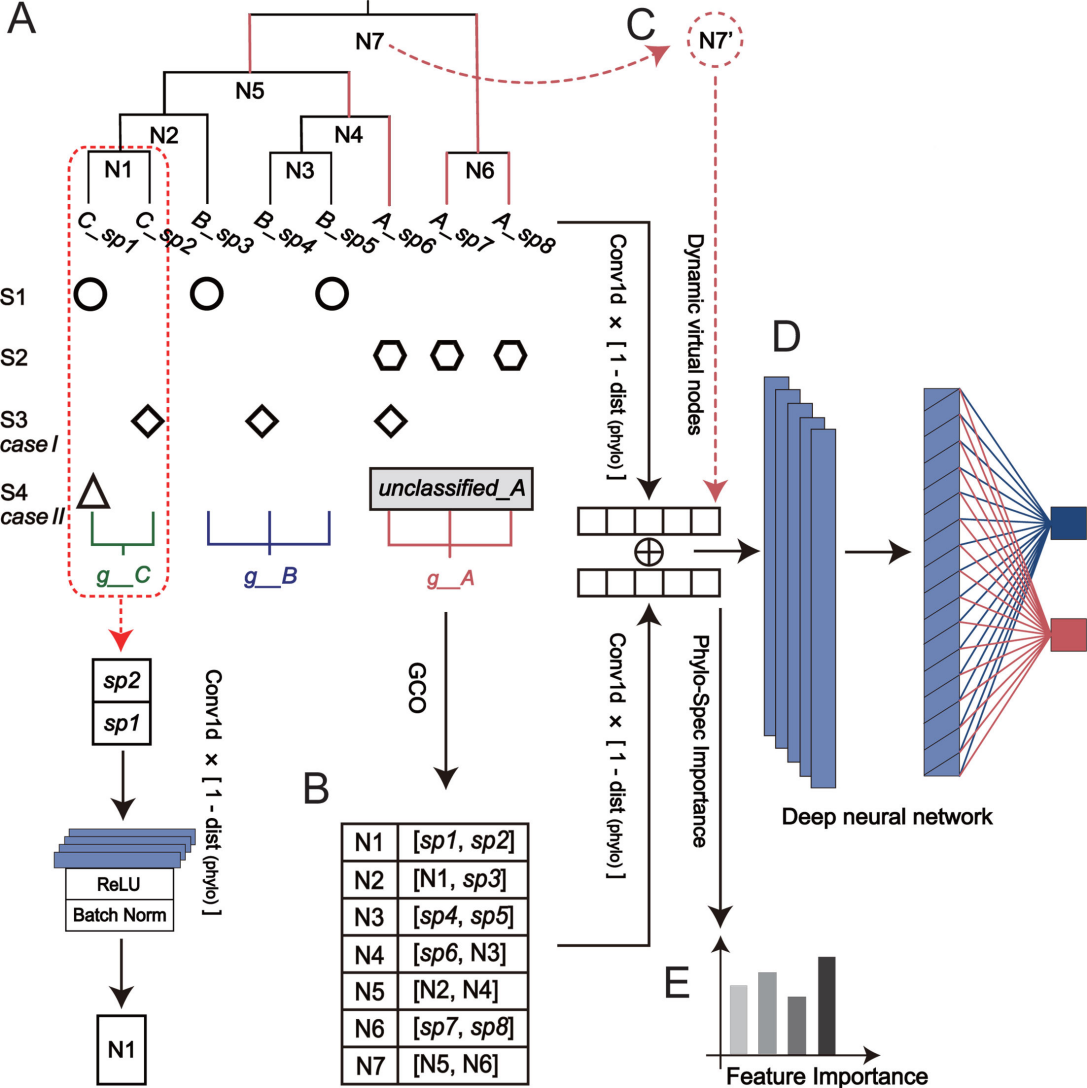
The overall framework of Phylo-Spec. (**A**) Group S1 and S2 are two groups with distinct microbes. In case I, although S3 shares tip nodes with S2, the overall community structure of S3 is more similar to S1 due to common phylogenetic branches. In case II, S4 contains the same tip node as S1, but it is considered as closer to S2 because they share more microbes under genus *g__A*. (**B**) To enable supervised learning employing phylogenetic information, Phylo-Spec identifies the parent-child relationship based on the phylogenetic topology by GCO (Get Convolution Order), and performs the one-dimensional convolution in a post-order traversal across the whole tree. (**C**) For unclassified species under genus *g__A*, Phylo-Spec dynamically places them on virtual nodes N7´ corresponding to branches under the same higher-level taxonomy (marked in red). (**D**) Finally, classification decisions are made by a deep neural network and a softmax function. (**E**) Phylo-Spec quantifies feature importance via information gain through tree propagation.

Microbiome data are inherently characterized by high dimensionality, overdispersion, and sparsity (16), often arising from natural microbial diversity or inaccuracies in profiling methods. To address these challenges, machine learning (ML) techniques have been widely employed to establish correlations between microbial dynamics and human health status (17–19). However, traditional ML approaches typically rely on microbial distribution patterns, which can be significantly affected by feature misalignment due to data heterogeneity, sequencing discrepancies, and annotation inconsistencies. For example, as illustrated in case I of Fig. 1A, when groups S1 and S2 are used for training, group S3 may be misclassified as S2 due to species overlap on *A_sp6*. Yet, a more detailed examination of the microbial relationships reveals that S3 is more likely to belong to S1 because of more shared phylogenetic branches, highlighting the limitations of relying solely on microbial distribution for classification.

Recently, deep learning (DL) methods have been developed to incorporate phylogenetic information into neural networks, such as PM-CNN (20), DeepPhylo (21), Ph-CNN (22), MetaDR (23), and TopoPhy-CNN (24). These approaches aim to better capture the relationships among species. However, MetaDR and TopoPhy-CNN rely on taxonomy trees rather than phylogenies, lacking quantitative representations of evolutionary distances across hierarchical levels and between microbes. While PM-CNN, DeepPhylo, and Ph-CNN do integrate phylogenetic structure into the modeling process, a persistent challenge is their inadequate handling of microbial taxa annotated at low-resolution levels (e.g., unclassified species from the LCA [lowest common ancestor] method in profiling). These unclassified species cannot be accurately positioned in the phylogenetic tree and are often excluded, potentially leading to biased classification outcomes. For example, in Fig. 1A, case II of group S4, which is more closely related to S2, may be misaligned with S1 if all unclassified species under genus *g__A* are discarded, resulting in inaccurate predictions. This highlights a major gap in existing models: none effectively address the impact of unclassified species or fully exploit the quantitative structure of phylogenetic trees (Table S1).

## MATERIALS AND METHODS

### Overall algorithm design

In this study, we propose Phylo-Spec, a phylogeny-driven deep learning model that integrates microbial phylogeny, richness, and taxonomy information for microbiome status detection and classification (Fig. 1). Phylo-Spec leverages the phylogenetic hierarchy of microbes to enhance feature fusion, providing a more robust classification model (Fig. 1A). Specifically, Phylo-Spec performs a post-order traversal of the phylogenetic tree to expand the convolutional features of microbial richness. These features are then integrated through an ensemble learning approach for classification decisions (Fig. 1B). This method significantly reduces misalignment caused by sparse data and inaccurate microbial profiling. Furthermore, to handle unclassified members of the microbiome—those that cannot be assigned to definite nodes in the phylogenetic tree—Phylo-Spec dynamically assigns these species to virtual ancestor nodes based on higher-level taxonomy, minimizing the impact of unclassified microbial data (Fig. 1C). Phylo-Spec quantifies the contribution of microbial features by recursively propagating their information gain across the phylogenetic tree (Fig. 1E). Thus, this approach enhances both model performance and interpretability, enabling the identification of key microbial patterns associated with different health states and offering valuable insights for clinical diagnosis.

### Post-order traversal convolution based on phylogeny topology

The microbial phylogeny is structured as a tree with pairwise parent-child relationships, where leaf nodes represent microbial taxa and internal nodes denote common ancestors, collectively reflecting species’ evolutionary lineages. Leveraging this structure, the Get Convolution Order (GCO) strategy (Algorithm 1) propagates information from leaves to root, effectively capturing multi-level phylogenetic dependencies. GCO further weights node relationships by phylogenetic distance, enabling closely related taxa to share richer contextual information and enhancing local structural representations. To capture the hierarchical dependencies, the framework devises multiple 1D convolutional filters specifically allocated to internal nodes to progressively enrich feature representations. Each convolution increases feature dimensionality and expands the receptive field, allowing integration of global, context-aware signals. Meanwhile, the model preserves the tree’s inherent local connectivity, enabling a unified representation of fine-grained lineage features and global evolutionary context.

Given a microbial relative abundance matrix in which all *N* samples are represented as an *N* × *S* matrix of abundance values across *S* microbial taxa (i.e., leaf nodes), we designed multiple convolutional operations to capture phylogeny-aware representations of microbial features. Specifically, for each leaf node, we compute a phylogenetically weighted abundance representation, enabling closely related taxa to share phylogenetic information more effectively throughout the convolutional hierarchy, as defined in equation 1:


(1) $$f_{\;}^{L}=\left\{f_{i}^{N\times 1}\times \left(1-{dist}_{phylo}\left(i\right)\right)\;|\;i\in 1,2,\ldots ,S\right\}$$

in which *f^L^* represents the set of phylogenetically weighted feature matrices for all leaf nodes, *f_i_^N^*^×1^ represents the phylogenetically weighted feature matrix of the *i*-th leaf node across all *N* samples. Each matrix has a dimensionality of *N* × 1, as each leaf node corresponds to a single taxonomic unit and thus represents only one feature dimension, and dist_(phylo)_ represents phylogenetic distance to the ancestor node.

By applying equation 1, we obtain the phylogenetically weighted feature matrices of the leaf nodes. Based on their evolutionary relationships, these initial features are then propagated upward to their common ancestors. For example, as illustrated in Fig. 1B, the features of *C_sp1* and *C_sp2* are aggregated at their shared ancestor node N1. This upward information flow is carried out via a post-order traversal of the phylogenetic tree, from leaf nodes to internal nodes, and finally to the root node, as formally defined in equation 2:


(2) $$f_{\;}^{I}=\left\{f_{i}^{N\times j}\times \left(1-{dist}_{phylo}\left(i\right)\right)\;|\;i\in 1,2,\ldots ,Root;\;j\in 1,2,\ldots ,S\right\}$$

in which *f^I^* represents the set of phylogenetically weighted feature matrices for all internal nodes, *f_i_^N^*^×^*^j^* represents the phylogenetically weighted feature matrix of the *i*-th internal node across all *N* samples. Each matrix has a shape of *N* × *j*, where *j* is the number of leaf nodes aggregated at the *i*-th internal node.

As illustrated in Fig. 1B, *C_sp1* and *C_sp2*, each with a matrix of shape (*N*, *1*), are first combined into an internal node N1 with a matrix of shape (*N*, *2*). Similarly, N1 is further aggregated with neighboring nodes to form N3, resulting in a matrix of shape (*N*, *3*). To preserve evolutionary constraints, we apply a distance-aware feature weighting mechanism, enabling closely related taxa to share more phylogenetic information throughout the convolutional hierarchy. This structured propagation framework enhances the receptive field adaptively and maintains local topological coherence, thereby improving robustness against feature misalignment and annotation inconsistencies.

### Algorithm 1. Get Convolution Order

**1: Input:** phylogenetic tree *# Input is a phylogenetic tree newick format*

**2: Output:** conv_order *# The output is a convolution order set table*

**3: conv_order** ← [] *# Initialize convolution order list*

**4: feature_map** ← {} *# Initialize feature map dictionary*

**5: all_features** ← [] *# List to store all features*

**6: function** postorder_traversal_and_conv(node)

**7: if** node.is_terminal() **then**

**8:** species ← node.name *# Get the name of the leaf node*

**9:** feature_map[species] ← get_leaf_feature(species) *# Get the feature of the leaf node*

**10:** all_features.append(feature_map[species]) *# Append the feature to all_features*

**11:** conv_order.append((species, “leaf”)) *# Record the convolution order for the leaf*

**12: return** feature_map[species] *# Return the feature of the leaf node*

**13: else**

**14:** children ← [] *# Store the child nodes of the current node*

**15:** child_feats ← [] *# Store the features of child nodes*

**16:** *# Traverse all child nodes of the current node*

**17: for** child in node **do**

**18:** child_feat ← postorder_traversal_and_conv(child) *# Recursively get the features of child nodes*

**19:** children.append(child.name) *# Collect the names of the child nodes*

**20:** child_feats.append(child_feat) **#** Add the child node features to child_feats

**21: end for**

**22:** *# Merge and convolve the child node features*

**23:** combined_feat ← merge_and_convolve(child_feats) *# Merge and convolve the child node features*

**24:** parent_feat ← assign_node_weight(combined_feat, node.name)

**25:** feature_map[node.name] ← parent_feat *# Store the feature of the parent node*

**26:** all_features.append(feature_map[node.name]) *# Append the parent node feature to all_features*

**27:** conv_order.append((node.name, “internal”)) *# Record the convolution order for the internal node*

**28: return** parent_feat *# Return the feature of the parent node*

**29: end if**

**30: end function**

**31:** postorder_traversal_and_conv(tree.root) *# Start post-order traversal and conv propagation*

**32:** combined_features ← combine_all_features(all_features) *# Combine all node features*

**33:** output ← activate_final_layer(combined_features) *# Get the final result through the output layer*

**34: return** output, conv_order # *Return the final output and the convolution order*

### Dynamic mapping of unclassified species in virtual nodes

The phylogeny-based feature convolution and fusion algorithm requires that all microbes in the community be assigned to definite nodes (e.g., tip nodes) in the phylogenetic tree. However, in real-world scenarios, some microbial species can only be annotated with low-resolution taxonomy. Due to discrepancies between phylogenetic and taxonomic hierarchies, these species cannot be mapped to any specific node in the phylogeny and are often excluded from further analysis. For instance, as shown in case II in Fig. 1, *unclassified_A* cannot be assigned to any tip node (e.g., species *A_sp6*, *A_sp7*, or *A_sp8*) due to the lack of species-level information, nor can it be linked to its common ancestor node N7, as this node also includes species from a different genus (*g__B*).

To address this, Phylo-Spec dynamically introduces virtual nodes during the feature convolution and fusion process, which are specifically designed to include only the branches under the same taxonomy (e.g., N7´ with branches marked in red; Fig. 1C). Meanwhile, the relative abundance of *unclassified_A* is assigned different weights across all species in the virtual species N7´. This approach allows unclassified or low-resolution species to be effectively incorporated into the phylogenetic framework without disrupting the integrity of the phylogeny. By mapping these unclassified species to virtual nodes, Phylo-Spec minimizes the potential bias introduced by missing or incomplete data, ensuring that these microbes do not interfere with the classification process. This technique preserves the overall structure of the phylogenetic hierarchy, enhancing the robustness of microbiome-based classification.

### Classification decision by deep neural network

Afterward, the leaf node features, internal node features, and root node features are integrated into a new feature matrix as in equation 3:


(3) $$f=f_{\;}^{L}⊕f_{\;}^{I}$$

which are then flattened into a single vector and interpreted by a deep neural network (i.e., fully connected layer; FC) for classification decisions. The FC also undergoes nonlinear enhancement through the ReLU function (25), and, finally, the softmax function (26) is utilized to generate classification results by producing a probability distribution for each category. To optimize model performance, this study implements a cross-entropy loss function, defined as $L_{binary}=-\frac{1}{n}\sum_{i=1}^{n}\left[y_{i}\times log\left(p_{i}\right)+\left(1-y_{i}\right)\times log\left(1-p_{i}\right)\right]$, is used for binary classification, $L_{multi}=-\frac{1}{n}\sum_{i=1}^{n}\sum_{k=1}^{K}y_{ik}\times log\left(p_{ik}\right)$, is used for multiple classifications, where *n* represents the number of samples, *y_i_* represents the true label of the *i*-th sample, *p_i_* represents the probability that the model predicts the positive class, *K* represents the number of classes, *y_ik_* indicates whether the *i-*th sample belongs to the *k*-th class, *p_ik_* represents the model predicts the probability that the *i*-th sample belongs to the *k*-th class. The model utilizes the Adam optimizer for efficient model parameter updates. Furthermore, we also set batch normalization (27) and dropout to 0.5 (28) in the model to improve convergence speed, enhance nonlinearity, and prevent overfitting.

### Importance scoring via propagated information gain in the phylogenetic tree

To enable model interpretability and quantify the phylogenetically informed contribution of each microbial feature, we compute the information gain at the root node and recursively propagate it through the tree to its descendants. As illustrated in Fig. 1, convolutional features are extracted from various nodes across the phylogenetic tree, including both tip nodes (e.g., *A_sp1*, *A_sp2*) and internal ancestral nodes (e.g., N1). The amount of information passed to each child node is inversely proportional to the length of the connecting phylogenetic branch, ensuring that closer evolutionary relationships receive more weight. The importance score of a node is defined as the sum of its own information gain and the propagated contributions from its ancestors, as formalized in equation 4:


(4) $$\mathrm{Imp}(n)=\mathrm{IG}(n)+\left\{ \begin{array}{rl} 0,\; & n=\text{ Root }\\ \mathrm{Imp}(n)\times \left(1-{\text{ dist }}_{\left(\text{phylo }\right)}\right),\; & n\neq \text{ Root } \end{array}\right.$$

in which *n* denotes a node, Imp*(n*) is the importance score of node *n*, IG*(n*) is the information gain value of node *n*, and dist_(phylo)_ is the phylogenetic distance to its ancestor node.

## RESULTS

### Experimental design

To evaluate the performance of Phylo-Spec in state classification, particularly its ability to capture phylogenetic relationships among microbes and incorporate unclassified species, we conducted a series of benchmark experiments using two *in silico* synthetic microbiome data sets and four real-world human gut microbiome data sets (Table 1; refer to “Simulation of artificial synthetic data sets,” below, for details).

**TABLE 1:** Information of microbiome data sets^*a*^

Data set	Type	Source and citation	Status	No. of microbes
Synthetic data set 1	Simulation	Simulation of case I by 60 species	Group S1 + S3, *n* = 77	60
			Group S2 + S4, *n* = 71	
Synthetic data set 2	Simulation	Randomly introducing 15%–20% unclassified species into synthetic data set one as case II	Group S1 + S3, *n* = 77	60
			Group S2 + S4, *n* = 71	
Amplicon data set 1	16S rRNA gene amplicon	PRJEB6070 (29)	Control, *n* = 268	1,495
		PRJNA280026 (29)	CRC, *n* = 144	
		PRJNA911189 (30)		
		PRJNA325650 (31)		
		PRJNA389927 (32)		
		PRJNA318004 (33)		
Amplicon data set 2	16S rRNA gene amplicon	PRJNA418765 (34)	Control, *n* = 406	416
		PRJNA450340 (35)	IBD, *n* = 1604	
		PRJNA317429 (36)		
		PRJEB13679 (6)		
		PRJEB13680 (6)		
		PRJEB42155 (37)		
		PRJEB23009 (38)		
		PRJNA313074 (39)		
WGS data set 1	Metagenome	ERP005534 (40)	Control, *n* = 74	354
		SRP136711 (41)	CRC, *n* = 67	
		PRJNA397112 (42)		
		PRJNA531273 (42)		
		PRJEB7774 (43)		
WGS data set 2	Metagenome	PRJNA422434 (44)	Control, *n* = 45	420
			T2D, *n* = 64	
Multi-status classification data set	16S rRNA gene amplicon	Amplicon data set 1	Control, *n* = 704	585
		Amplicon data set 2	CRC, *n* = 142	
		PRJNA544721 (29)	IBD, *n* = 914	
		PRJNA282013 (45)	IBS, *n* = 84	
		PRJNA578223 (46)	ASD, *n* = 138	
		PRJNA589343 (46)		

^*a*^ WGS, whole-genome sequencing.

Synthetic data set 1 comprises 148 samples of human gut microbiome data, artificially simulated to represent microbial distributions of S1, S2, and their misaligned neighboring microbes, as depicted in case I (e.g., S3 is a misaligned version of S1; Fig. 1A). This data set was synthesized from 60 human gut microbial species. Synthetic data set 2 was then generated by randomly converting 15%–20% of the taxa from synthetic data set 1 into unclassified species, as in case II, which are annotated only at the genus level.

We also assessed Phylo-Spec’s performance on real-world microbiomes produced by 16S rRNA gene amplicon sequencing and whole-genome sequencing (WGS). Each type of data corresponds to two data sets with different healthy statuses (Table 1). Sequence profiling for 16S and WGS data was performed using Parallel-Meta Suite (47) and MetaPhlAn4 (48), respectively (refer to “Materials and data sets,” below for details about data preprocessing).

For each data set, we performed fivefold cross-validation, splitting 80% of the data for training and 20% for testing in each iteration. To alleviate the data imbalance, we applied the SMOTE (49) method to the training set. We compared Phylo-Spec to other commonly used ML and DL approaches in microbiome studies, including Random Forest (RF), a regular Convolutional Neural Network (CNN), and phylogeny-aware models such as PM-CNN, MetaDR, and DeepPhylo (refer to “Model implementation and configuration,” below for details). The overall performance was evaluated using AUC (area under the receiver operating characteristic curve). We ensured that the training process does not involve any information from the testing phase, thereby preventing label leakage.

### *In silico* synthetic microbiomes confirmed the efficacy of algorithm design

**Fig 2:**
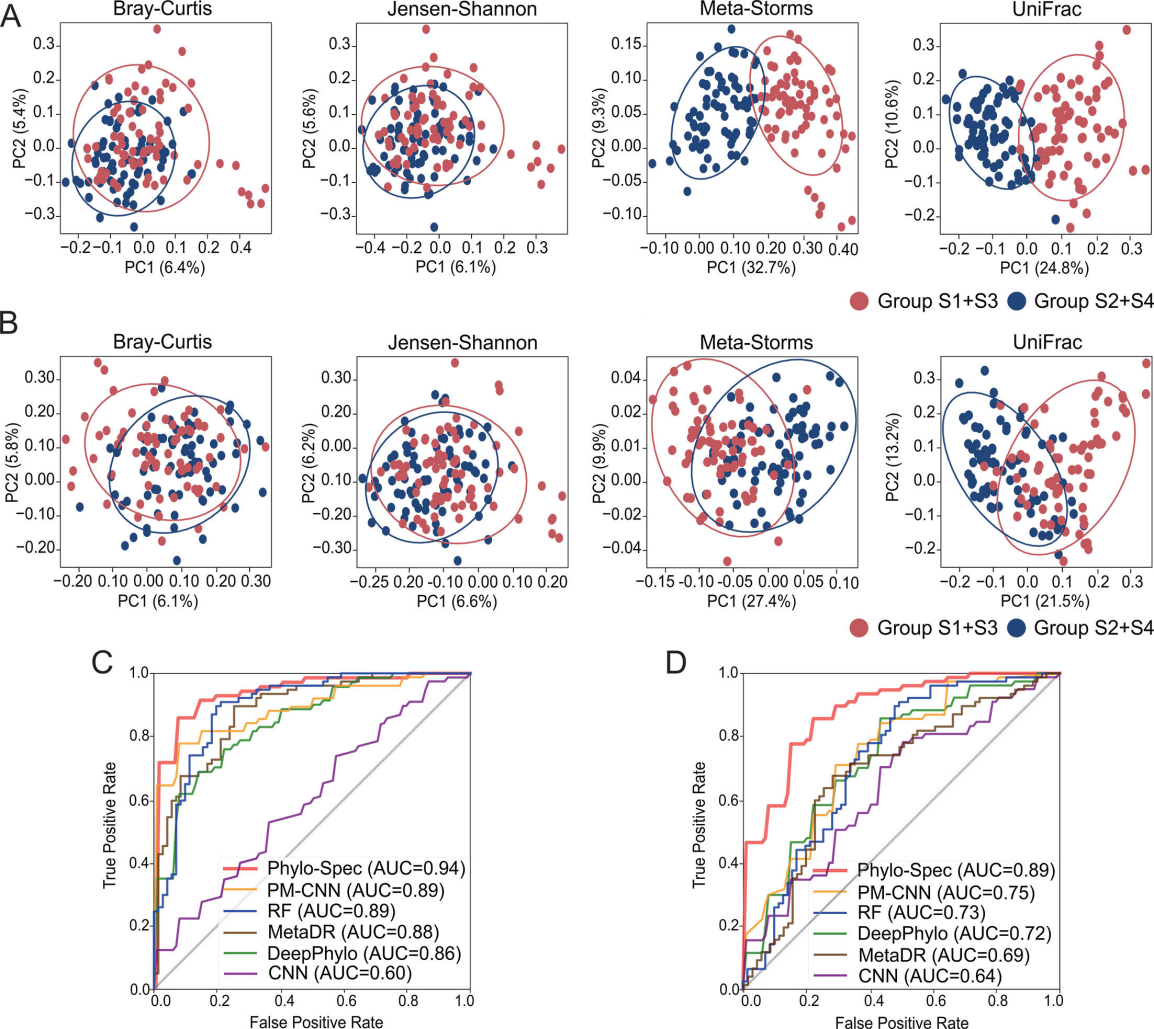
Performance of classification on synthetic data sets. (**A**) PCoA patterns of synthetic data set 1. Circles denote 90% confidence ellipses, similarly hereinafter. (**B**) PCoA patterns of synthetic data set 2. (**C**) AUC of classification on synthetic data set 1. (**D**) AUC of classification on synthetic data set 2. Source data are available in Data sheets S1 and S2.

On synthetic data set 1, we first conducted beta-diversity analysis using both traditional distance metrics, such as Bray-Curtis and Jensen-Shannon, as well as the phylogeny-based Meta-Storms (50) and UniFrac distance (51). The principal coordinate analysis (PCoA) patterns highlighted the critical role of phylogeny in capturing the structural differences between microbial communities of the two groups (Fig. 2A), which was further supported by the PERMANOVA test (Table 2). As anticipated, Phylo-Spec achieved the highest AUC of 0.94 (Fig. 2C), followed by two other phylogeny-aware models, PM-CNN and DeepPhylo, which surpassed the traditional models, such as RF and CNN.

**TABLE 2:** PERMANOVA test results of synthetic data sets

Distance metrics	Bray-Curtis	Jensen-Shannon	Meta-Storms	UniFrac
Synthetic data set 1				
*P*	0.001	0.001	0.001	0.001
*F*	3.755	2.953	47.882	31.863
*R*^2^	0.105	0.019	0.621	0.179
Synthetic data set 2				
*P*	0.001	0.001	0.001	0.001
*F*	2.458	2.179	21.205	15.272
*R*^2^	0.017	0.015	0.127	0.100

In the presence of unclassified species that could not be assigned to specific phylogenetic nodes (synthetic data set 2), beta-diversity patterns were significantly disrupted (Fig. 2B), leading to decreased precision across all other models. However, by applying the dynamic mapping strategy for unclassified species, Phylo-Spec maintained a high AUC of 0.89 (Fig. 2D), demonstrating its robust performance in handling sparse data and unclassified species.

### Application on amplicon sequencing data of human microbiome

**Fig 3:**
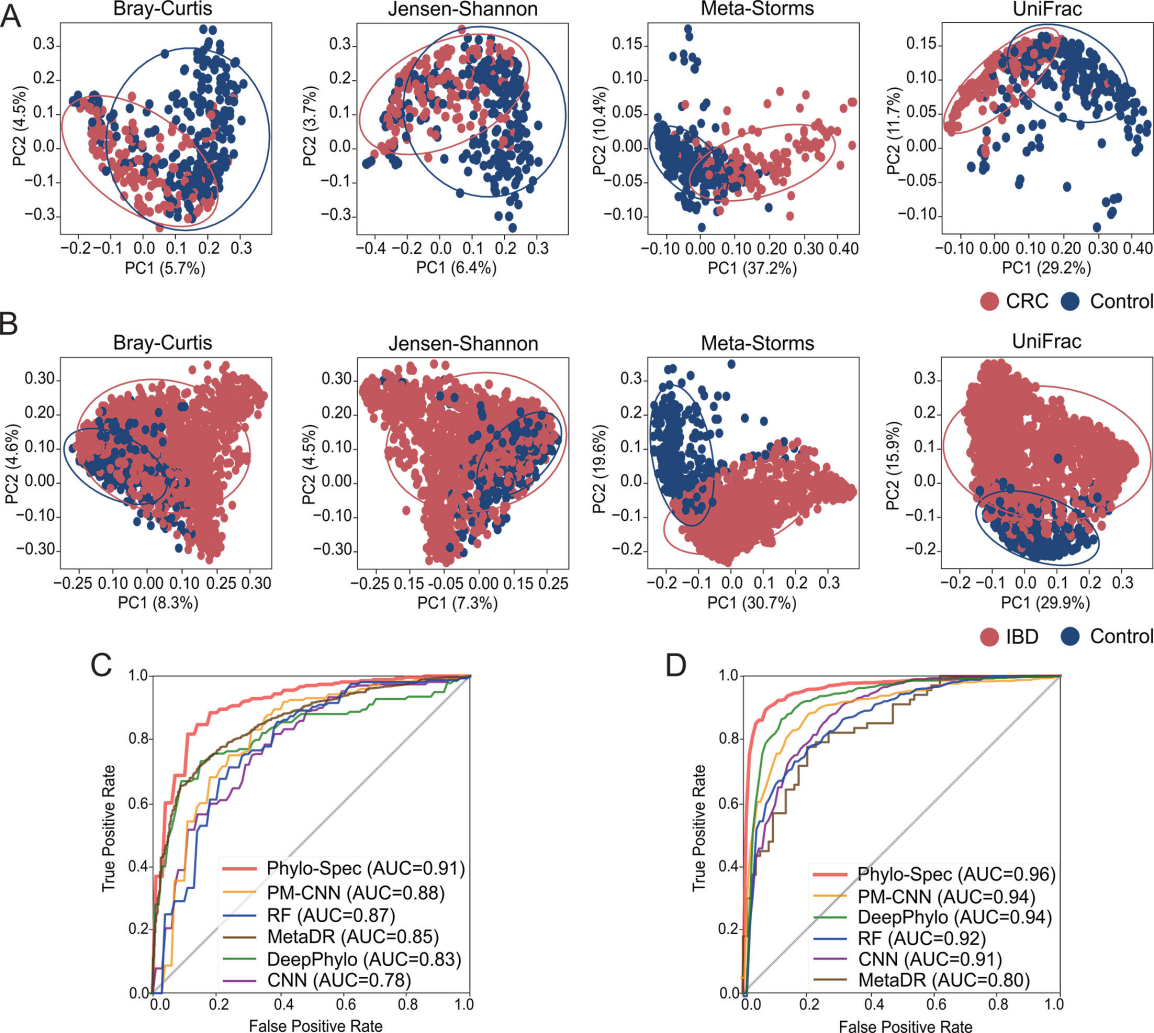
Performance of state detection on amplicon sequencing data sets. (**A**) PCoA patterns of amplicon data set 1 with CRC. (**B**) PCoA patterns of amplicon data set 2 with IBD. (**C**) AUC of state detection on amplicon data set 1 with CRC. (**D**) AUC of state detection on amplicon data set 2 with IBD. Source data are available in Data sheets S3 and S4.

Next, we applied Phylo-Spec to two real amplicon sequencing data sets (amplicon data set 1 and amplicon data set 2; Table 1) focused on IBD and colorectal cancer (CRC). Previous studies have highlighted the impact of the microbiome on these diseases, with significant differences in beta-diversity between healthy and diseased states (Fig. 3A and B). After ASV (amplicon sequence variant) denoising, most short reads were successfully mapped to a reference database and the pre-built phylogenetic tree.

With the added advantage of phylogeny-based information fusion, Phylo-Spec achieved the highest disease detection accuracy (Fig. 3C and D), reinforcing the value of phylogenetic context in microbiome-based health state detection.

### Application on metagenomic sequencing data of human microbiome

**Fig 4:**
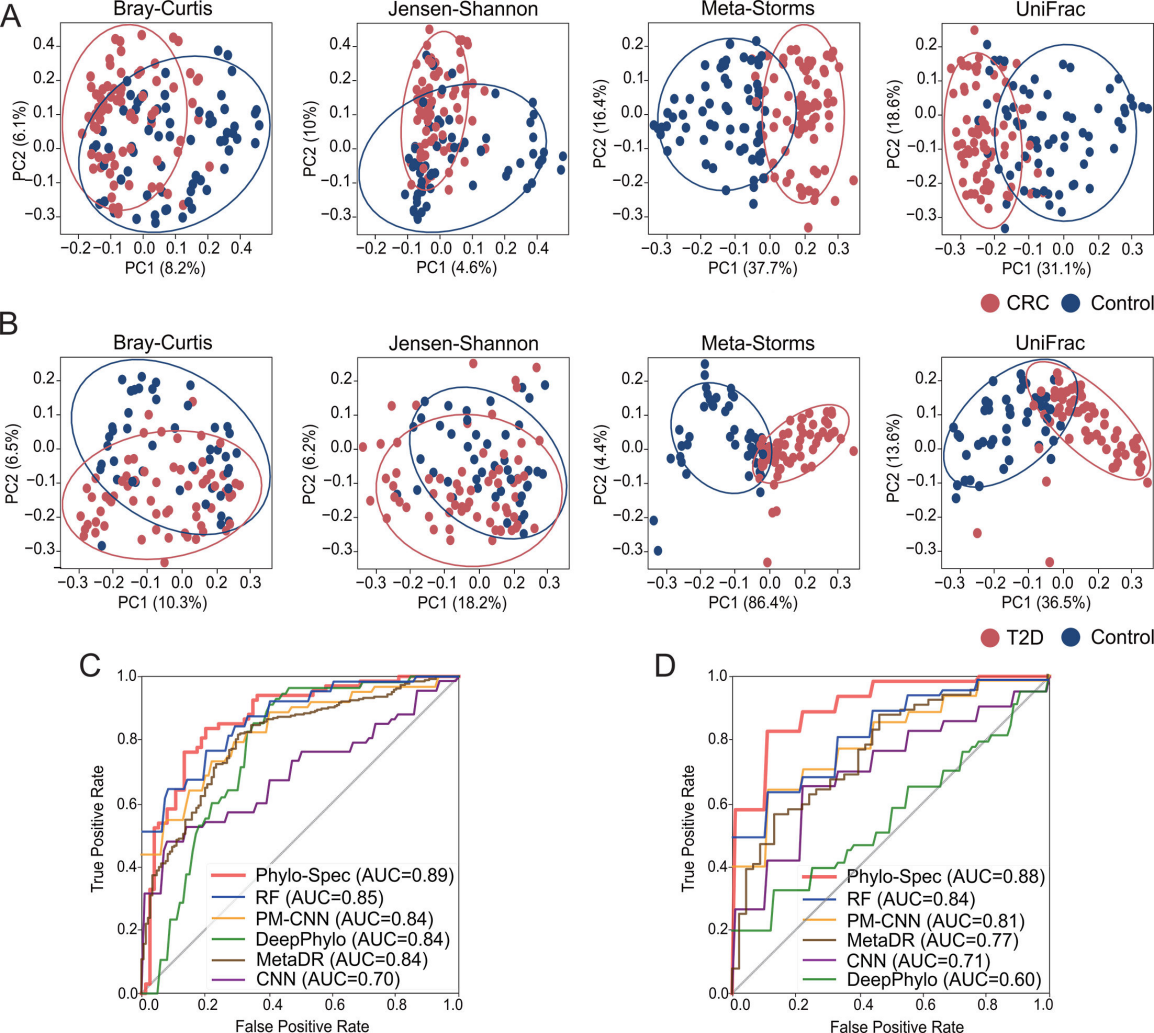
Performance of state detection on metagenomic sequencing data sets. (**A**) PCoA patterns of WGS data set 1 with CRC. (**B**) PCoA patterns of WGS data set 2 with T2D with 90% confidence ellipses. (**C**) AUC of state detection on WGS data set 1 with CRC. (**D**) AUC of state detection on WGS data set 2 with T2D. Source data are available in Data sheets S5 and S6.

Here, we further validated the performance of Phylo-Spec on two metagenome data sets collected from IBD and T2D studies (WGS data set 1 and WGS data set 2; Table 1). Metagenomic sequencing provides species-level resolution for deciphering microbiome structures, but the resulting profiles often contain a significant proportion of unknown microbes annotated only at the genus or family level. These unclassified species are typically excluded in diversity analyses and subsequent modeling, leading to a bias in understanding and employing the variation in microbial diversity (Fig. 1A, 4A and B).

Through its dynamic node mapping, Phylo-Spec fully leveraged this incomplete annotation information, achieving a high overall AUC of 0.89 and 0.88 on the two WGS data sets, respectively (Fig. 4C and D). This performance outpaced other models, highlighting the model’s ability to incorporate unclassified or incomplete microbial data effectively.

### Multi-status classification in human microbiome

**Fig 5:**
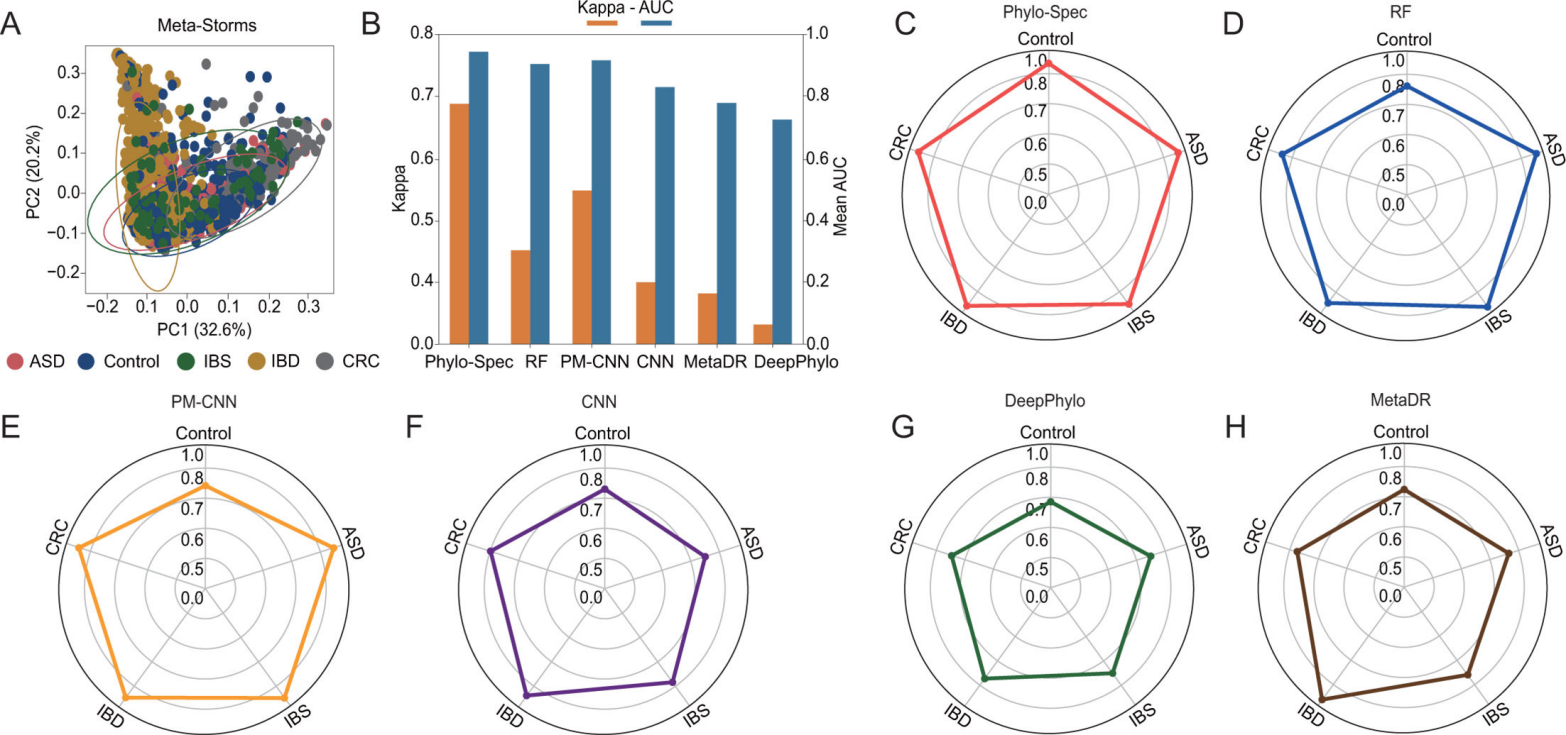
Performance of multi-status classification across different models. (**A**) PCoA patterns of multi-status classification data set. (**B**) AUC and Kappa values for each model. (**C–H**) AUC of each status across different approaches. Source data are available in Data sheet S7.

To comprehensively assess the performance of Phylo-Spec in diverse clinical contexts, we evaluated its multi-class classification capability using 16S rRNA gene amplicon data sets across five disease categories: CRC, IBD, autism spectrum disorder (ASD), and irritable bowel syndrome (IBS), along with healthy controls (multi-status classification data set; Table 1). The performance of five representative models was compared based on average AUC and Cohen’s Kappa values using fivefold cross-validation, ensuring robustness across different data partitions (Fig. 5).

The results reveal that RF performs well in distinguishing specific diseases like ASD and IBS but tends to misclassify healthy samples, leading to high false-positive rates akin to random guessing (Fig. 5C). DeepPhylo, while incorporating phylogenetic distances, is primarily designed for binary classification, and its performance in a multi-class setting is moderate overall—particularly poor for ASD (Fig. 5F). PM-CNN improves upon traditional CNN by embedding evolutionary relationships, showing better accuracy in disease classification (Fig. 5D and E), but still falls short of Phylo-Spec, likely due to its inability to handle unclassified microbial taxa effectively. Despite comparable average AUC and Kappa scores among the models, Phylo-Spec consistently delivers the most balanced and effective multi-class performance across all disease categories (Fig. 5A).

Therefore, even under conditions of beta-diversity disruption in multi-disease settings (Fig. 5A), Phylo-Spec significantly enhanced downstream disease prediction and classification performance. This underscores Phylo-Spec’s strength as a robust and phylogenetically informed framework for microbiome-based health status recognition and disease prediction, paving the way for more integrative and interpretable approaches in microbiome research.

### Assessment of feature importance promotes both the performance and interpretability

Here, we applied the Phylo-Spec Importance scoring to both synthetic and real-world data sets. This approach not only helped to understand the key microbial features driving classification but also further improved classification accuracy by elaborated features.

To comprehensively evaluate the effectiveness and stability of the Phylo-Spec Importance method, we adopted a rigorous experimental design. The data set was randomly partitioned into 10 equal parts, of which 7 were used for training and the remaining 3 for testing. Importantly, Phylo-Spec Importance scores were derived solely from the training sets without involving any operations—such as data preprocessing, feature selection, or normalization—that could potentially introduce data leakage. This ensures that the importance scores reflect purely the intrinsic signal from the training data, free from any information contamination from the test sets.

After computing the Phylo-Spec Importance scores from the training data, we observed that the selected features led to consistently good predictive performance on the held-out test sets. To further assess the robustness of our feature selection strategy and the stability of the importance rankings, we performed a cumulative feature evaluation. Specifically, we ranked all features by their Phylo-Spec Importance scores and then incrementally added features—starting from the top-ranked one up to the full set—and calculated the AUC at each step.

This procedure was repeated five times randomly, minimizing the influence of stochastic variation (e.g., due to data shuffling or model randomness). The standard error of the AUC across these runs was consistently below 0.02, suggesting that the performance gains attributed to Phylo-Spec Importance-based feature selection are both reproducible and statistically robust.

**Fig 6:**
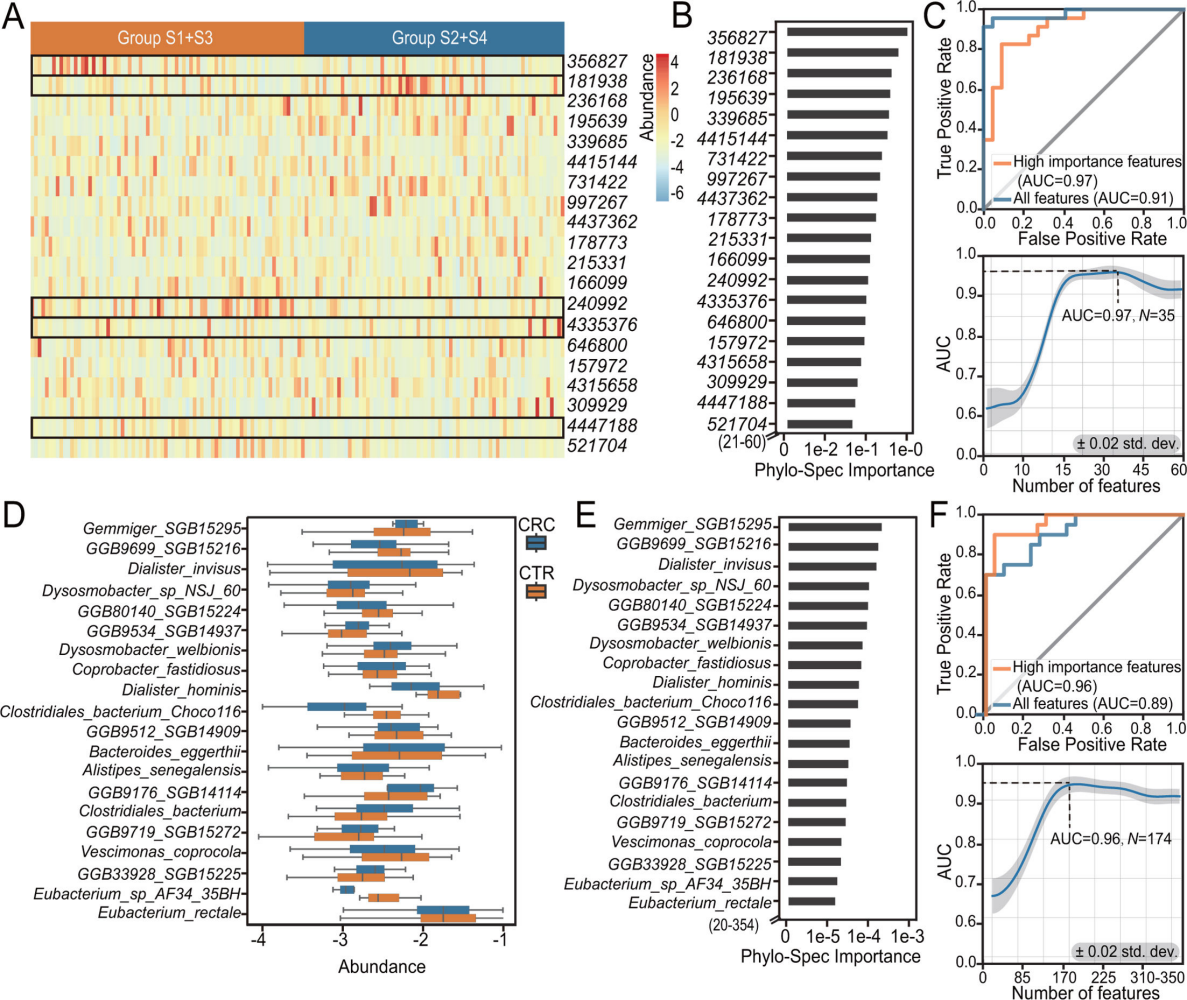
Validation of important features in metagenomic sequencing data set and synthetic data set. (**A**) Heatmap of the top-ranked 20 features in synthetic data set 1. The numbers represent OTU (operational taxonomic unit) identifiers in Greengenes (v.13-8), similarly hereinafter. (**B**) Phylo-Spec Importance of the top 20 features in synthetic data set 1. (**C**) AUC curve of the top *N* features in synthetic data set 1, showing how AUC changes with the number of features. (**D**) Box plot of significant features among the top 20 features in WGS data set 1 with CRC. The letters represent species names in MetaPhlAn4, similarly hereinafter. (**E**) Phylo-Spec Importance of the top 20 features in WGS data set 1 with CRC. (**F**) AUC curve of the top *N* features in WGS data set 1 with CRC, showing how AUC changes with the number of features. Source data are available in Data sheets S8 and S9.

In synthetic data set 1, feature selection based on Phylo-Spec Importance closely aligned with the experimental design. Among the top 20 ranked features, many of the intentionally modified taxa between the two groups were consistently identified (e.g., 356827, 181938, and 240992, highlighted by the black boxes in Fig. 6A and B). We further assessed model performance by conducting classification using only the top-ranked features (Fig. 6A and B). An optimal AUC of 0.97 was achieved using 35 features, exceeding the original AUC of 0.91 produced by unfiltered features, after which the performance plateaued (Fig. 6C). This finding suggests that a relatively small subset of highly informative features can drive optimal classification, reducing model complexity while enhancing interpretability.

To validate the utility of Phylo-Spec Importance in real-world data, we also applied the same approach to the CRC metagenomic data set (WGS data set 1; Fig. 6D and E). AUC analysis across varying numbers of top-ranked features revealed that classification performance peaked at an AUC of 0.96 with 174 features and then stabilized (Fig. 6F), surpassing the previous model of all features. These results further confirm the robustness of Phylo-Spec Importance in identifying significant microbial features, even in complex intestinal environments.

## DISCUSSION

In this study, we introduced Phylo-Spec, a novel phylogeny-driven deep learning model designed to improve microbiome status classification by integrating microbial phylogeny, richness, and taxonomy. Through extensive benchmarking with both synthetic data sets and real-world microbiome data sets, Phylo-Spec demonstrated superior performance in capturing the complex relationships within microbial communities and maintaining robustness in the presence of unclassified species.

The results highlighted Phylo-Spec’s ability to significantly outperform existing machine learning and deep learning approaches. By dynamically assigning unclassified species to virtual nodes based on higher-level taxonomy, Phylo-Spec was able to minimize feature misalignment caused by data sparsity and improve classification accuracy in diverse microbiome data sets. In addition, Phylo-Spec not only enhances the interpretability of the model by quantifying the contributions of both ancestral and tip nodes, but also enables the identification of a minimal set of highly informative microbial features for accurate prediction. This is particularly valuable in clinical contexts, where understanding the microbial drivers of disease can support targeted diagnostics and therapeutic strategies.

Overall, Phylo-Spec provides a promising framework for microbiome-based health status identification and disease prediction, with great potential for both research and clinical applications. It is capable of handling high-dimensional, sparse, and incomplete microbiome data while leveraging phylogenetic information, making it a powerful tool to advance microbiome diagnostics and precision medicine. Nevertheless, it should be noted that Phylo-Spec may not be universally applicable to all scenarios. Future work will focus on further optimizing its scalability, validating its performance in broader contexts, and integrating it into more comprehensive microbiome-based health-monitoring and diagnostic platforms.

### Materials and data sets

The data sets for testing are listed in Table 1. Profiling of 16S rRNA amplicon data sets was performed by Parallel-Meta Suite (47). Amplicon sequences were denoised by ASV and clustered based on these denoised ASVs, and similar sequences were clustered into OTUs (operational taxonomic units) and mapped to the Greengenes (v.13-8) (52) reference database and phylogeny tree. Metagenome sequences (WGS) were preprocessed by MetaPhlAn4 (48), keeping only bacterial profiles, and the specific version used was mpa_vOct22 with the CHOCOPhlAnSGB reference database, released in December 2022.

To address data sparsity, we excluded microbial features with zero sequence counts in more than 90% of the samples, without further processing of low-abundance species. We retained these species, as we believe doing so helps capture global microbial relationships through phylogeny and reduces the risk of feature misplacement. We also applied ComBat (53) batch correction for real data sets that contain multiple data sources to minimize technical variation.

### Simulation of artificial synthetic data sets

To evaluate the robustness of classifiers under complex microbiome conditions, we constructed two simulated data sets (Table 1). For synthetic data set 1, based on the empirical abundance distribution of the 2,000 random human gut microbiomes in Microbiome Search Engine (54), we selected 60 highly prevalent OTUs to construct a core feature set and defined two microbial community structures with distinct phylogenetic differences, corresponding to group S1 + S3 (*n* = 77) and group S2 + S4 (*n* = 71) (Fig. S1). At the same time, the phylogenetic tree used in the simulation data is the reference tree from Greengenes (v.13-8).

To simulate common sources of random variation in microbiome studies, we added zero-mean Gaussian noise with a standard deviation of 1 × 10^−6^ to all samples in both groups. This perturbation reflects natural stochastic fluctuations in microbial abundance profiles. To further mimic systematic technical biases—such as those arising from sequencing platform effects, batch effects, or inconsistencies in taxonomic annotation—we introduced abundance misalignment. Specifically, 15%–20% of randomly selected OTUs were perturbed in a random 20%–30% of the samples (i.e., group S3) within group S1, whereby the abundance of selected OTUs was misassigned to their phylogenetically neighboring OTUs, thereby introducing feature misalignment. To maintain biological plausibility and ensure data integrity, all perturbed abundance values were subject to non-negativity truncation, with negative values set to zero. The resulting perturbed and subsequently normalized abundance matrices comprise synthetic data set 1 (Fig. S1A), which was used to evaluate classification robustness under conditions of feature misalignment.

Building on this, we constructed synthetic data set 2 to evaluate the capability of Phylo-Spec in handling unannotated or unknown species (i.e., group S4, derived from a random 20%–30% of group S2 samples; Fig. 1A). Specifically, we randomly selected 15%–20% of the 60 core species and simulated them as “unclassified species” with only genus-level annotation (Fig. S1B). However, if all unclassified species within the genus *g__A* are excluded during analysis, samples from S4 may be erroneously assigned to S1 due to loss of critical discriminatory features. In fact, group S4 is more closely related to group S2. This design mimics a common limitation of LCA-based methods when facing incomplete reference databases or low-resolution sequences and allows us to examine whether Phylo-Spec can still perform accurate classification based solely on phylogenetic structure.

### Model implementation and configuration

We compared Phylo-Spec with RF, CNN, and other state-of-the-art approaches, including MetaDR, DeepPhylo, and PM-CNN. Initially, all models were evaluated using their default parameters, and if the performance was found to be unsatisfactory, key hyperparameters were further tuned through grid search or manual adjustment. Notably, all experiments were conducted on the same data set without the use of any external data. To ensure consistency and fairness, all models followed the same preprocessing procedure, as detailed in “Materials and data sets,” above. The parameters for each model were tuned as shown in Table 3.

**TABLE 3:** Hyperparameter combinations for model tuning

Machine learning models	Implementations	n_estimators (no. of trees)		max_depth (depth of trees)		max_features (no. of features sampled at each split)					
RF	Python 3.8 Torch ≥ 2.3.1 Pandas ≥ 2.2.2 Numpy ≥ 1.26.4 Scikit-learn ≥ 1.4.2 Imbalanced-learn ≥ 0.12.3 Ete3 ≥ 3.1.3 Matplotlib ≥ 3.7.2 Biopython ≥ 1.83	{100, 200, 500}		{4, 5}		{log_2_(# of features), 0.5$\sqrt{\#\;of\;features},\sqrt{\#\;of\;features}$}					
Deep learning models		Learning rate	Weight decay	Dropout	Activation	Kernel size	Hidden size	# of epochs	Batch size	# of conv layers	# of dense layers
CNN		{1e – 2, 1e-3, 1e – 4}	{0.0, 1e–2, 1e-3, 1e–4}	{0.02, 0.03, 0.05}	{ReLU, Tanh, Sigmoid}	{3, 5, 7}	{32, 64, 128}	{2, 4, 6, 8, 10}	{256, 128, 64}	{3, 4}	{2, 3}
MetaDR				{0.02, 0.03, 0.05}		{3, 5, 7}	{500}		Image conversion and full batch	{2}	{2}
DeepPhylo				{0.02, 0.03, 0.05}		{3, 5, …, 15}	{32, 64, 128}		{256, 128, 64}	{1}	{1}
PM-CNN				{0.02, 0.03, 0.05}		{2, 4, 6, 8}	{32, 64, 128}		{256, 128, 64}	{6, 9}	{2, 3}
Phylo-Spec				{0.02, 0.03, 0.05}		{1, 3, 5, 7}	{32, 64, 128}		{256, 128, 64}	Adapted to phylogenetic trees	{3}

## Data Availability

Phylo-Spec was developed using the Python-based PyTorch framework (55). The source code, usage guidelines, and additional resources are available in the GitHub repository at https://github.com/qdu-bioinfo/Phylo-Spec. All source data are made available in the supplemental material, the input data are accessible in the GitHub repository at https://github.com/qdu-bioinfo/Phylo-Spec/tree/main/data, source codes of all experiments are available in the GitHub repository with detailed usages at https://github.com/qdu-bioinfo/Phylo-Spec/tree/main/multi_models, and the reference databases are also available in the GitHub repository at https://github.com/qdu-bioinfo/Phylo-Spec/tree/main/database.

## SUPPLEMENTAL MATERIAL

Supplemental Material msystems.01453-25-s0001.docx Fig. S1 and Table S1.

Data sheets S1 to S11 msystems.01453-25-s0002.xlsx Source data for all figures.

ASM does not own the copyrights to Supplemental Material that may be linked to, or accessed through, an article. The authors have granted ASM a non-exclusive, world-wide license to publish the Supplemental Material files. Please contact the corresponding author directly for reuse.
