# Supplementary material for: Phylo-Spec: a phylogeny-fusion deep learning model advances microbiome status identification
Source: mSystems. 2025 Nov 24;10(12):e01453-25. doi: 10.1128/msystems.01453-25 (PMC12710343; doi:10.1128/msystems.01453-25)
Supplement: Supplemental Material — Fig. S1 and Table S1. [file msystems.01453-25-s0001.docx]

**Supplementary Materials for “Phylo-Spec: a phylogeny-fusion deep learning model advances microbiome status identification”**

**Supplementary Figure**


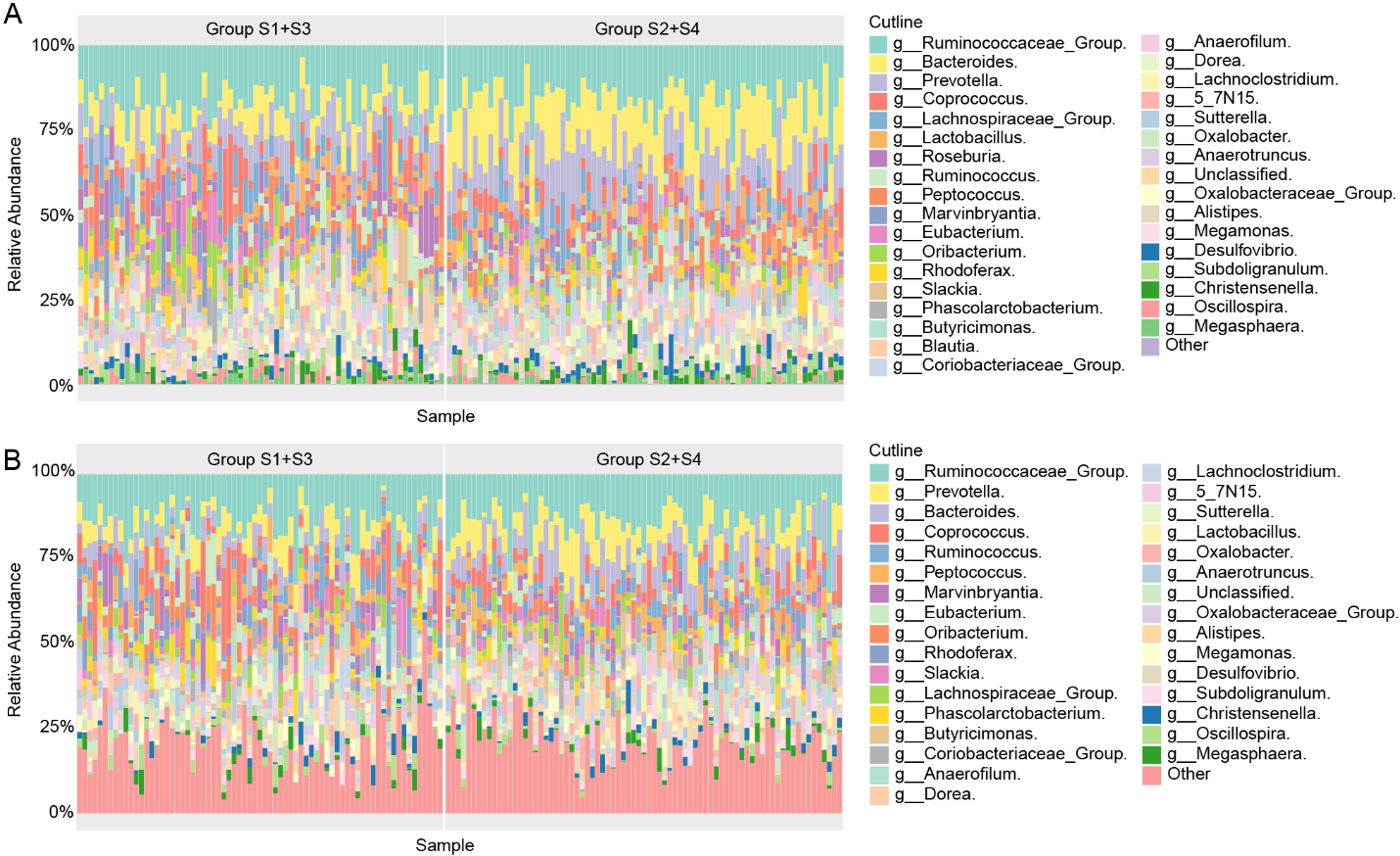


**Fig. S1. The distribution bar chart of the synthetic dataset at the genus-level.** (A) Distribution of Synthetic Dataset 1. (B) Distribution of Synthetic Dataset 2 with unclassified taxa. Source data is available in Supplementary Datasheet 10 and Datasheet 11.

**Supplementary Table**

**Table S1. Comparison of state-of-the-art models for microbiome research.**

|  | **Phylogeny used** | **Phylogeny validated** | **Feature misalignment addressed** | **Unclassified taxa handled** | **Interpretability** | **Multi-disease classification** |
| --- | --- | --- | --- | --- | --- | --- |
| **TopoPhy-CNN** | **×** | **×** | **×** | **×** | **×** | **×** |
| **MetaDR** | **×** | **×** | **×** | **×** | **√** | **×** |
| **DeepPhylo** | **√** | **×** | **×** | **×** | **×** | **×** |
| **Ph-CNN** | **√** | **×** | **×** | **×** | **×** | **×** |
| **PM-CNN** | **√** | **×** | **×** | **×** | **×** | **√** |
| **Phylo-Spec** | **√** | **√** | **√** | **√** | **√** | **√** |
